# Morning Drams

**Published:** 1882-05

**Authors:** 


### MORNING DRAMS.

If there is one form of "drinking" more injurious than others it is that which consists in the frequent recourse to drams at odd times between meals. That there is a great deal of this sort of tippling in vogue cannot be doubted, when we take cognizance of the very large and, as it would appear, the increasing number of young men and even women of respectable appearance who are to be met in the streets of London or any large city as early as noon already to an evident degree under the influence of an intoxicant. Discounting the multitude of such inebriated persons or habitual debauchees, and those who drink so deeply at night that they retain the effects of the poison until late in the following day, it is still only too plain that a considerable proportion of the staggering and half-unconscious or unduly excited individuals about are the victims of the morning dram. It is a serious question whether public houses should be allowed to begin the day before noon. It is surely unnecessary that workmen and workwomen should commence their potations earlier than the usual dinner-hour. As it is, no sooner have the bricklayers, painters, plumbers, plasterers, or carpenters engaged in the repair of a house returned from their breakfast and arranged their tools than they go or send for beer. The result of this early beginning of the drink-business is that before the afternoon has well set in they are apt to be practically useless or only able to labor with a great effort for self-control. While the doors of public houses stand open those who have money will enter and buy drink. Perhaps if the purveyors of intoxicants were not at liberty to commence their dangerous trade until just before the first meal in the day at which stimulants are legitimately taken, there would be a less common use of the "morning dram," one of the most mischievous "drinks" in which the multitude, especially the young, can possibly indulge. —*London Lancet.*

